# Duration of Enterovirus D68 RNA Shedding in the Upper Respiratory Tract and Transmission among Household Contacts, Colorado, USA

**DOI:** 10.3201/eid2911.230947

**Published:** 2023-11

**Authors:** Hai Nguyen-Tran, Careese Thompson, Molly Butler, Kristen R. Miller, Laura Pyle, Sarah Jung, Shannon Rogers, Terry Fei Fan Ng, Janell Routh, Samuel R. Dominguez, Kevin Messacar

**Affiliations:** University of Colorado, Aurora, Colorado, USA (H. Nguyen-Tran, K.R. Miller, L. Pyle, S.R. Dominguez, K. Messacar);; Children’s Hospital Colorado, Aurora (C. Thompson, M. Butler, S. Jung);; Centers for Disease Control and Prevention, Atlanta, Georgia, USA (S. Rogers, T.F.F. Ng, J. Routh)

**Keywords:** Enterovirus D68, EV-D68, acute flaccid myelitis, viruses, respiratory infections, virus shedding, natural history, Colorado, USA

## Abstract

Enterovirus D68 (EV-D68) causes cyclical outbreaks of respiratory disease and acute flaccid myelitis. EV-D68 is primarily transmitted through the respiratory route, but the duration of shedding in the respiratory tract is unknown. We prospectively enrolled 9 hospitalized children with EV-D68 respiratory infection and 16 household contacts to determine EV-D68 RNA shedding dynamics in the upper respiratory tract through serial midturbinate specimen collections and daily symptom diaries. Five (31.3%) household contacts, including 3 adults, were EV-D68–positive. The median duration of EV-D68 RNA shedding in the upper respiratory tract was 12 (range 7–15) days from symptom onset. The most common symptoms were nasal congestion (100%), cough (92.9%), difficulty breathing (78.6%), and wheezing (57.1%). The median illness duration was 20 (range 11–24) days. Understanding the duration of RNA shedding can inform the expected rate and timing of EV-D68 detection in associated acute flaccid myelitis cases and help guide public health measures.

Enterovirus D68 (EV-D68) is a nonpolio enterovirus that causes respiratory illness and can result in acute flaccid myelitis (AFM), a devastating polio-like neurologic disease (*1*–*17*). This positive-sense single-stranded RNA virus is transmitted primarily through respiratory droplets and is an emerging pathogen of public health concern. EV-D68 has been recognized as a cause of recurrent cyclical outbreaks of asthma-like respiratory disease and AFM since 2014, affecting families, healthcare systems, and society (*1*,*2*,*5*,*8*,*10*,*11*,*13*,*18*,*19*).

The causal link between EV-D68 and AFM is strong, but EV-D68 is still not identified in most epidemiologically linked AFM cases (*3*,*9*,*17*). Compared with poliovirus and other nonpolio enteroviruses, EV-D68 has properties that more closely resemble rhinovirus (*9*,*13*). EV-D68 can be acid labile and is thus less likely to be detected in stool, grows most optimally at 33°C, and is transmitted in the upper respiratory tract (*9*,*13*). However, unlike rhinoviruses and some other respiratory viruses, the duration of RNA shedding in the upper respiratory tract and the timeline of clinical manifestations of EV-D68 respiratory illness are unknown.

In this study, we investigated the duration of EV-D68 RNA shedding in the upper respiratory tract and the associated clinical characteristics in children hospitalized with EV-D68 respiratory disease and their household contacts. We hypothesized that the duration of RNA shedding of EV-D68 would be similar to that of rhinovirus at a median of 11.4 days (*20*). Determining RNA shedding dynamics has implications for expected rates of detection in EV-D68–associated AFM cases that can help inform clinical diagnosis and public health measures.

## Methods

### Study Description

We performed a prospective observational cohort study during September–November 2022, during an EV-D68 outbreak at Children’s Hospital Colorado in Aurora, Colorado, USA. Children’s Hospital Colorado is a large, freestanding children’s hospital with 444 beds serving a 7-state region. The study was approved by the Colorado Multiple Institutional Review Board. We obtained written informed consent, and assent when applicable, from all participants.

### Study Participants and Study Procedures

Patients hospitalized at Children’s Hospital Colorado for respiratory illness who were positive for rhinovirus/enterovirus by provider-directed testing on the BioFire Respiratory 2.1 Panel (BioFire Diagnostics, https://www.biofiredx.com) were eligible to be included as primary participants in the study. Household contacts of enrolled primary participants were also eligible for inclusion. We excluded persons <2 months of age or >65 years of age, persons who tested positive for SARS-CoV-2 (for infection control purposes), and persons with contraindications to respiratory specimen collection.

For primary participants and household contacts who were present at bedside, we collected a midturbinate flocked swab sample (COPAN Diagnostics, https://www.copanusa.com) on the day of enrollment, daily while the primary participant was admitted, and every 3 days after discharge until 21 days after enrollment. Midturbinate swab specimens were collected by study personnel or by the participant, parent, or legal guardians (who were trained by study personnel) during hospital admission and by the participant, parent, or legal guardian during home collections. For household contacts who were not present at the hospital, midturbinate swab samples were collected every 3 days by the participant or a parent or guardian from day of enrollment until 21 days after enrollment.

We collected demographic and patient history by using a standardized questionnaire and verified data through the electronic medical record. We asked participants to recall symptoms, medications, and interventions experienced during the 14 days before enrollment using standardized lists. Prospective symptom diaries including the same list of symptoms, medications, and interventions were completed daily until 21 days after enrollment by the participant, parent, or legal guardian. Assuming a 20% censoring rate and a hypothesized mean duration of 11.4 days, we planned to enroll a minimum of 30 participants to produce a 2-sided 80% CI width of 6.3 days.

### Samples and Laboratory Testing

Midturbinate swab samples were stored in PrimeStore Molecular Transport Media (Longhorn Vaccines and Diagnostics, https://www.lhnvd.com), which was previously validated by our laboratory for home collection and storage conditions for EV-D68 qualitative detection (*21*). Samples were stored in a refrigerator, delivered from home after the 21-day collection period, then aliquoted and frozen at −80°C until testing.

An aliquot from each collection day from all participants underwent EV-D68 real-time qualitative reverse transcription PCR testing. We extracted total nucleic acid, including exogenously added internal positive control DNA (TaqMan Exogenous Internal Positive Control Reagents; ThermoFisher Scientific, https://www.thermofisher.com) on the QIAGEN EZ1 Advanced XL platform using the Virus 2.0 Mini Kit (https://www.qiagen.com), then performed cDNA synthesis on the ABI Veriti Thermal Cycler platform using TaqMan Reverse Transcription Reagents (ThermoFisher Scientific). We added 5 μL of cDNA to a final PCR reaction volume of 20 μL with the reaction component qScript XLT One-Step RT-qPCR ToughMix Low-Rox (Quantabio, https://www.quantabio.com), primers and probes at a final reaction concentration of 250 nM, and internal positive control primers and probe (ThermoFisher Scientific) (*22*,*23*). We performed reverse transcription PCR on the ABI 7500 Real-Time PCR System (ThermoFisher Scientific) with the following cycling conditions: 45°C for 10 minutes, then 95°C for 10 minutes, followed by 45 cycles of 95°C for 15 seconds and 60°C for 1 minute. We considered a cycle threshold (Ct) value <40 positive for EV-D68. This laboratory-developed protocol for EV-D68 detection was validated in-house and found to have a limit of detection of 700 genome copies/mL and 100% overall agreement (46/46) with an independent comparator assay specific to EV-D68 (T.F.F. Ng, unpub. data, https://www.biorxiv.org/content/10.1101/2022.10.06.511205v2). We performed further sequencing on an EV-D68–positive specimen using an established enterovirus typing method (*24*) to identify the EV-D68 subclade in the study.

### Statistical Analysis

**Figure 1:**
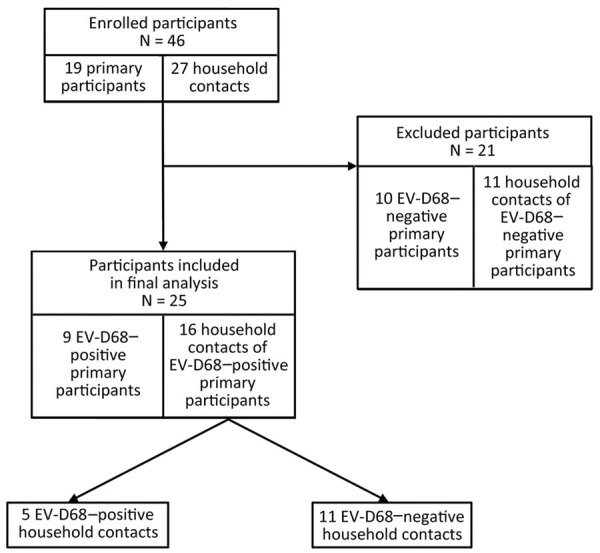
Study flowchart showing selection process of study participants included in the analysis of EV-D68 RNA shedding in the upper respiratory tract and associated clinical characteristics, Colorado, USA. EV, enterovirus.

We included primary participants who tested positive for EV-D68 and their household contacts in the analysis (Figure 1). We summarized demographics, medical histories, medications, and interventions using frequency and percentage or median, interquartile range, and range.

We determined the duration of RNA shedding of EV-D68–positive participants from symptom onset to time-of-negativity, defined as the first negative result that was immediately followed by a subsequent negative result. Participants were censored on the last day a specimen was collected that followed the study schedule if they did not complete further collections per the study schedule. We created a Kaplan-Meier curve for RNA shedding for all EV-D68–positive participants and further stratified it by children (<18 years of age) and adults. We used log-rank tests to compare time-to-negativity by group and calculated median and 95% CI for RNA shedding duration from the Kaplan-Meier curve. Because of the small sample size and few events at later time points, the upper limits of the 95% CIs of median survival times were undefined, so the range was also reported from the raw data. We calculated symptom duration from the first day until the last day the symptom was reported during the study period and illness duration from first day of any symptom to last day of any symptom. For each symptom and illness duration, we calculated median and interquartile range for day of onset and day of offset. We used R version 4.2.2 (The R Foundation for Statistical Computing, https://www.r-project.org) for analysis.

## Results

During September–November 2022, we enrolled 46 participants (19 primary participants and 27 household contacts) in the study. Of the primary participants, 9 (47.4%) were EV-D68–positive; no other codetections were detected on respiratory pathogen panel. We included the 9 EV-D68–positive primary participants and their 16 household contacts (n = 25) in the analysis (Figure 1). We confirmed EV-D68 subclade B3 by sequencing in a study participant, which is consistent with the predominant clade circulating in the United States during 2022 (*25*,*26*).

### Study Population Characteristics

For the 9 primary participants, the median age was 2.2 years (range 8 months–17 years); 7 (77.8%) were boys and 2 (22.2%) were girls (Table 1). The age range of all household contacts was 4 months–44 years (Table 1). Of the 16 household contacts, 5 (31.3%) persons, each from a different household, were positive for EV-D68. Of the EV-D68–positive household contacts, 2 were sibling children (<5 years) and 3 were mothers of the primary participants. Prematurity, chronic lung disease, and chronic heart disease were reported among the primary participants; only 1 participant (11.1%) reported a history of asthma (Table 2). Of the EV-D68–positive household contacts, 2 (40%) of 5 reported a history of asthma; both were mothers of primary participants.

**Table 1:** Demographic characteristics of participants and number of specimens collected in study of EV-D68 RNA shedding in the upper respiratory tract and associated clinical characteristics, Colorado, USA*

Characteristic	All participants, N = 25	EV-D68–positive primary participants, n = 9	EV-D68–positive household contacts, n = 5†	EV-D68–negative household contacts, n = 11
Age, y				
Median (IQR)	17.0 (2.2–35.0)	2.2 (1.5–3.3)	32.0 (4.0–39.3)	34.5 (26.2–39.2)
Range	0.3–44.9	0.7–17.0	0.7–44.9	0.3–40.2
Sex				
F	15 (60.0)	2 (22.2)	4 (80.0)	9 (81.8)
M	10 (40.0)	7 (77.8)	1 (20.0)	2 (18.2)
Race				
American Indian or Alaska Native	0	0	0	0
Asian	0	0	0	0
Black or African American	0	0	0	0
Native Hawaiian or other Pacific Islander	0	0	0	0
White	25 (100)	9 (100)	5 (100)	11 (100)
Ethnicity				
Hispanic or Latino	15 (60)	6 (66.7)	3 (60)	6 (54.5)
Not Hispanic or Latino	10 (40)	3 (33.3)	2 (40)	5 (45.5)
Median household size (IQR)‡				
Overall, all members	6 (5–7)	6 (5–7)	6 (5–7)	6 (5–6)
Household members >18 y	2 (2–2)	2 (2–2)	2 (2–2)	2 (2–2)
Household members <18 y	3 (2–4)	3 (3–4)	3 (3–4)	4 (2–4)
No. midturbinate specimens collected per participant during study period				
Median (IQR)	9 (7–10)	9 (8–11)	9 (9–14)	8 (7–10)

*Values are no. (%) except as indicated. EV, enterovirus; IQR, interquartile range. †Two EV-D68–positive household contacts were children and had onset of symptoms starting 2 d before or on the same day as the primary participant. The other 3 EV-D68–positive household contacts were adults and had onset of symptoms that started 3–5 d after the primary participant. ‡Including the primary participant.

**Table 2:** Selected medical history of study participants in study of EV-D68 RNA shedding in the upper respiratory tract and associated clinical characteristics, Colorado, USA*

Comorbidity†	No. (%) participants			
	All participants, N = 25	EV-D68–positive primary participants, n = 9	EV-D68–positive household contacts, n = 5	EV-D68–negative household contacts, n = 11
Asthma	4 (16.0)	1 (11.1)	2 (40.0)	1 (9.1)
History of prematurity	3 (12.0)	2 (22.12)	1 (20.0)	0
Chronic heart disease	1 (4.0)	1 (11.1)	0	0
Chronic lung disease	1 (4.0)	1 (11.1)	0	0
Other reported history‡	4 (16.0)	2 (22.2)	1 (20.0)	1 (9.1)
None reported	15 (60.0)	5 (55.6)	1 (20.0)	9 (81.8)

*EV, enterovirus. †Comorbidities are not mutually exclusive. ‡Other includes Trisomy 21, chromosomal abnormality, developmental delay, cavernous malformation, obesity, hypothyroidism, or nephrolithiasis.

### Medications, Interventions, and Hospitalization

All 9 primary participants received albuterol, antipyretics, and supplemental oxygen support (Table 3), and 7 (77.8%) received steroids. Seven (77.8%) primary participants required noninvasive positive pressure ventilation support during their admission, and 1 (11%) required intubation. Both EV-D68–positive household contacts who were children subsequently required hospital admission and supplemental oxygen support but did not require noninvasive positive pressure ventilation or intubation (Table 3). None of the adult EV-D68–positive household contacts required hospital admission, although 1 person sought outpatient care.

**Table 3:** Medications and interventions received during the study period for participants in study of EV-D68 RNA shedding in the upper respiratory tract and associated clinical characteristics, Colorado, USA*

Intervention	All participants, N = 25	EV-D68–positive primary participants, n = 9	EV-D68–positive household contacts, n = 5	EV-D68–negative household contacts, n = 11
Medications				
Albuterol	13 (52.0)	9 (100)	3 (60.0)	1 (9.1)
Antipyretic	17 (68.0)	9 (100)	3 (60.0)	5 (45.5)
Steroids	8 (32.0)	7 (77.8)	1 (20.0)	0
No. outpatient visits during study period†				
0	13 (52.0)	1 (11.1)	2 (40.0)	10 (90.9)
1	11 (44.0)	7 (77.8)	3 (60.0)	1 (9.1)
2	1 (4.0)	1 (11.1)	0	0
Hospital course				
Required admission	11 (44.0)	9 (100)	2 (40.0)	0
Required ICU stay	8 (32.0)	8 (88.9)	0	0
Median length of stay (IQR), d	4.0 (3.0–6.5)	5.0 (4.0–7.0)	2.0 (1.5–2.5)	NA
Median ICU length of stay (IQR), d	2.5 (1.8–4.3)	2.5 (1.8–4.3)	NA	NA
Oxygen support at any time during admission				
Supplemental oxygen	11 (44.0)	9 (100)	2 (40.0)	0
Noninvasive positive pressure ventilation	7 (28.0)	7 (77.8)	0	0
Intubation	1 (4.0)	1 (11.1)	0	0

*Values are no. (%) except as indicated. ICU, intensive care unit; IQR, interquartile range; NA, not applicable. †Includes clinic, urgent care, and emergency department visits.

Of the 11 children admitted with EV-D68 respiratory disease (9 primary participants and 2 household contacts), the median length of stay was 4 days (range 1–26 days). Eight (72.7%) required intensive care unit (ICU) admission; median ICU stay was 2.5 days (range 1–17 days). The most common discharge diagnosis was asthma exacerbation/reactive airways (45.5%), followed by viral lower respiratory tract infection (bronchiolitis/pneumonitis) (36.3%); a superimposed bacterial pneumonia was noted in 18.2% (not mutually exclusive).

### EV-D68 RNA Shedding and Associated Characteristics

**Figure 2:**
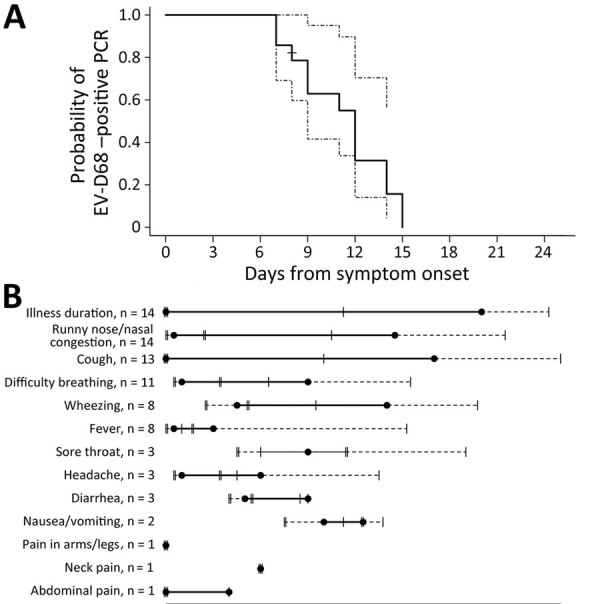
EV-D68 RNA shedding curve (A) and associated symptoms (B) for all EV-D68–positive participants (n = 14), Colorado, USA. A) Dotted line on Kaplan-Meier curve represents 95% CI. The + mark indicates the time at which 1 participant was censored at the last day of inpatient collection because they did not report dates of home testing. B) Number of participants reporting the symptom at any time is indicated. Black dots represent the median onset and median offset time for each symptom; solid horizontal line represents the duration between median onset and median offset time. The double vertical hash lines represent the 25th and 75th quartile for onset time, and the single vertical hash lines represent the 25th and 75th quartile for offset time. Symptoms on standardized list were abdominal pain, back pain, cough, diarrhea, difficulty breathing, difficulty swallowing, difficulty walking, facial droop/weakness, fever, headache, muscle jerks/tremors, nausea/vomiting, neck pain, pain in arms/legs, runny nose/nasal congestion, sore throat, vision changes, weakness in arms/legs, and wheezing. EV, enterovirus.

**Figure 3:**
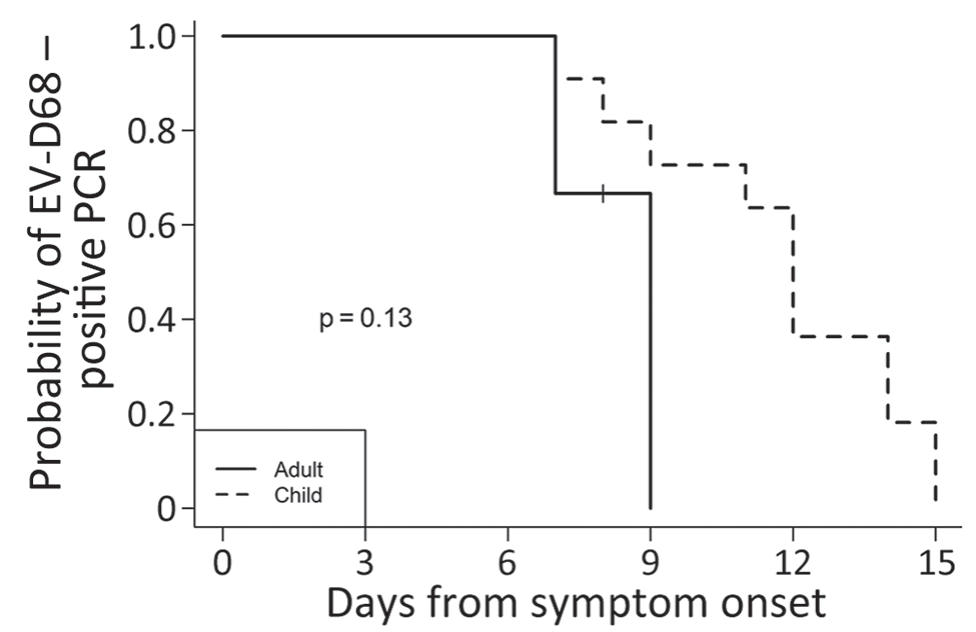
EV-D68 RNA shedding curve for adults (n = 3) versus children (n = 11) in study of shedding and household transmission, Colorado, USA. All children required hospitalization. The + mark indicates the time at which 1 participant was censored at the last day of inpatient collection because they did not report dates of home testing. The log-rank test statistic was used to test whether the 2 curves were different. EV, enterovirus.

We analyzed RNA shedding of EV-D68 in the upper respiratory tract for all 14 EV-D68–positive participants (Figure 2). One participant was censored at the last day of inpatient collection because dates of home testing were not reported. The median duration of RNA shedding from illness onset was 12 days overall (range 7–15 days; 95% CI lower limit 9, upper limit undefined [because of small sample size; see Methods]). For adults, median duration was 9 days (range 7–9 days; 95% CI lower limit 7, upper limit undefined), and for children, median duration was 12 days (range 7–15 days; 95% CI lower limit 11, upper limit undefined; p = 0.13) (Figure 3). By 9 days after illness onset, 25% of participants were no longer shedding detectable RNA; by 14 days, 75% no longer had detectable EV-D68 RNA.

The most common reported symptom in EV-D68–positive participants was runny nose/nasal congestion (100%), followed by cough (92.9%), difficulty breathing (78.6%), wheezing (57.1%), and fever (57.1%). Less common symptoms included sore throat (21.4%), headache (21.4%), and diarrhea (21.4%). The median illness duration was 20 days (range 11–24 days); cough had the longest median duration (18 days, range 7–30 days), followed by runny nose and nasal congestion (15 days, range 3–30 days) and wheezing (12 days, range 3–26 days). Twelve (85.7%) of the 14 EV-D68–positive participants were still symptomatic after they were no longer shedding detectable RNA. AFM did not develop in any EV-D68–positive participants in the study.

All EV-D68–positive household contacts were symptomatic. The adult household contacts reported mild respiratory symptoms, such as cough, runny nose and nasal congestion, wheezing, fever, and sore throat. Onset of symptoms in household contacts ranged from 2 days before to 5 days after symptom onset in the primary participant.

## Discussion

We found that EV-D68 RNA shedding duration is more similar to that of rhinoviruses than of other enteroviruses; the median duration of EV-D68 RNA shedding in the upper respiratory tract was 12 days. Understanding RNA shedding dynamics can inform the expected rate at which EV-D68 could be detected in the upper respiratory tract at various timepoints in the course of illness, particularly in EV-D68–associated AFM cases. Insight into the natural history of EV-D68 from this study also helps inform transmission risk among household members and further defines the range of disease severity and symptoms, which can guide public health measures and management.

Cases of AFM are often preceded by a respiratory or febrile illness; median delay is 5–7 days from prodromal respiratory or febrile illness to onset of neurologic symptoms (*1*,*2*,*27*). Delayed recognition and misdiagnosis of AFM can result in even further delays in specimen collection and testing, leading to many cases going undiagnosed until weeks to months after symptom onset (*28*). Enterovirus PCR detection in sterile sites (e.g., cerebrospinal fluid [CSF] or blood) is specific for the diagnosis of enterovirus neurologic disease, but detection at those sites in AFM is exceedingly rare; enteroviruses are more commonly detected in nonsterile sites, particularly the respiratory tract (EV-D68) and stool (poliovirus, enterovirus A71) (*29*). Unfortunately, in many AFM cases, the recommended set of biospecimens is collected late in the course of disease, if at all, and samples from nonsterile sites, particularly the respiratory tract, are often lacking (*29*). In a 2014 study of AFM cases in the United States, 120 AFM cases were reported, but a respiratory sample was submitted for only 56 (46%) patients. In patients for whom respiratory samples were tested for enterovirus/rhinovirus, 44% were collected >12 days after symptom onset, and our findings suggest that at least half would no longer have been shedding detectable EV-D68 RNA. Those early data demonstrating that the proportion of EV-D68–positive samples increased when samples were collected closer to symptom onset (47% positive for EV-D68 if tested <7 days from onset of respiratory or febrile illness) hinted at the importance of early sample collection and prompted this effort to learn more about shedding dynamics (*29*).

Our study confirms that timely specimen collection, particularly of respiratory specimens, is critical for detecting EV-D68 in suspected AFM cases (*20*,*30*), whereas other enteroviruses can shed for weeks in stool. On the basis of RNA shedding curve in the upper respiratory tract of this study, all participants had detectable EV-D68 RNA at 5 days after symptom onset, reinforcing the goals of prompt recognition and early sampling at the time of neurologic symptom onset in cases of suspected AFM. In EV-D68–associated AFM cases in which recognition is delayed, this study suggests that, by 9 days after prodromal symptom onset, 25% will no longer be shedding detectable RNA; 50% will no longer be shedding by 12 days, and 75% by 14 days. The later a respiratory specimen is collected in the course of AFM, the less likely it is that EV-D68 will be detected; by 14 days after prodromal symptom onset, EV-D68 is exceedingly unlikely to be detected, even if causing disease. Providers should be encouraged to collect respiratory samples, in addition to stool, CSF, and serum samples, as soon as AFM is suspected on physical examination, without waiting for confirmatory imaging, lumbar puncture, or classification and confirmation by public health authorities (*31*). Furthermore, this study highlights that, in some AFM cases, EV-D68 RNA detection might not be possible if recognition or sample collection is delayed. That finding speaks to the need for complementary diagnostics, such as EV-D68 antibody testing in CSF samples, to detect intrathecal antibody production as the serologic footprint of neurologic infection when viral RNA is no longer detectable, which has become the standard for diagnosing West Nile virus and other neuroinvasive arboviruses in immunocompetent hosts (*32*).

Enrolling household contacts in this study, including adult family members with mild disease, also provided insight into EV-D68 transmission and epidemiology outside of the hospital setting, where it has been primarily studied. Of the 9 households in the study, 5 (55.6%) had EV-D68–positive household contacts, suggesting transmission within households occurs between children and parents. All EV-D68–infected persons were symptomatic. We found no evidence of asymptomatically infected persons shedding RNA to suggest the potential for silent transmission, although brief shedding or transmission before or after the collection period could not be excluded. Additional community studies are needed to further elucidate the burden of EV-D68 respiratory disease.

Both parents and siblings of children hospitalized with EV-D68 were themselves infected with EV-D68, but the severity of disease between adult and child contacts differed. The EV-D68–infected adults had only mild respiratory symptoms and did not require hospitalization, whereas the 2 EV-D68–infected child siblings had more severe respiratory illness that required hospitalization (*15*). Despite similar exposures, not all household contacts were infected, some had mild disease, and others had more severe disease, suggesting a role for host immunity in infection. Studies have found that, by the time they reach school age, most persons have neutralizing serum antibodies against EV-D68 suggestive of previous infection (*33*–*35*). This study demonstrated that adults, who would be expected to have neutralizing antibodies, still could be infected with EV-D68 and experience respiratory symptoms, but their symptoms tended to be milder; the median duration of RNA shedding was 9 days after symptom onset. Thus, having systemic immunity from previous infection might be protective against more severe manifestations but might not provide sterilizing immunity to prevent infection entirely or protect against mild respiratory disease. Future studies should investigate the role of innate and adaptive mucosal and systemic immunity in the development of different EV-D68 disease manifestations (e.g., asymptomatic, mild to severe respiratory disease, or neurologic disease). Clarifying the host immune response to natural EV-D68 infection will be key to developing effective monoclonal antibodies and vaccine candidates for treatment and prevention.

In terms of symptoms, we found that EV-D68 infection caused prolonged illness, including wheezing lasting nearly 2 weeks. As for rhinoviruses, EV-D68 causes upper respiratory symptoms and triggers bronchoreactive symptoms in the lower respiratory tract (*7*). Most children with EV-D68 respiratory illness in this study were hospitalized with asthma-like bronchoreactive symptoms and received treatment with medications typically used for asthma, such as albuterol and steroids, which are generally not recommended for viral respiratory infections in young children. However, unlike rhinovirus-associated exacerbations occurring predominantly in children with underlying asthma, only 1 child with EV-D68 in our study had asthma. Previous studies have found that EV-D68 induces interleukin-17–dependent airway inflammation and hyperresponsiveness, which might explain the response to bronchodilators and steroids in children without underlying asthma seen in our study and in previous EV-D68 outbreaks (*36*–*38*). Long-term longitudinal studies and a better understanding of the mechanisms by which EV-D68 causes respiratory disease are needed to determine optimal management and whether early EV-D68 infection predisposes patients to develop asthma in the future, similar to other respiratory viruses, such as respiratory syncytial virus (*39*).

The first limitation of our study was the limited time frame in which participants were enrolled, given the small window during which EV-D68 circulation peaks; thus, our sample size was small and might not be fully generalizable to a broader population. Provider-directed respiratory pathogen panel testing in hospitalized children might have biased the primary participant study population toward more severe illness; however, we also enrolled household contacts to capture a broader range of clinical manifestations. We infer that the RNA shedding of EV-D68 in the upper respiratory tract, particularly in those with mild respiratory illness, might be similar to the shedding that precedes EV-D68 neurologic manifestations (i.e., AFM). However, that possibility would be extremely challenging, if not impracticable, to investigate without enrolling AFM patients before neurologic disease develops, during the prodromal respiratory/febrile illness. The results of this study cannot be used to evaluate the infectious period of EV-D68 because we relied on detection of viral RNA above our assay’s limit of detection and did not perform viral culture because of the difficulty of growing EV-D68 caused by its atypical pH and temperature requirements (*13*). However, because RNA is typically shed longer than live culturable virus, this study provides upper limits for the longest potential period of infectiousness, which could help inform infection control measures. We spaced sample collection to every 3 days after discharge for ease of home collection, but that timing might have decreased the precision of shedding duration endpoints after hospitalization; conservatively calculating duration from the first negative sample rather than the last positive might slightly overestimate shedding durations. Finally, we used midturbinate swab specimens (instead of nasopharyngeal swabs) to detect RNA shedding, and some of those specimens were collected by the patient or a parent or legal guardian. Nasopharyngeal swab specimens are often used as the standard for upper respiratory pathogen detection, and use of midturbinate swab specimens in this study might have decreased sensitivity; however, midturbinate swab specimens are increasingly being used in practice because of their tolerability and were more practical for this study given the serial nature of sample collection. In addition, although we cannot definitively ensure the quality of home-collected samples, and differences between home-collected samples and those collected by study personnel might exist, studies have shown that parent-collected or self-collected specimens are adequate for pathogen detection and are a feasible convenient option, particularly in studies collecting serial samples (*40*–*42*).

Given current epidemiologic patterns, future EV-D68 outbreaks of respiratory disease and AFM are likely. The knowledge generated by this study about RNA shedding, transmission dynamics, and the natural history of EV-D68 can help families, providers, and healthcare systems anticipate frequency of illness, expected disease course, and resource needs to prepare for outbreaks. We found a median duration of EV-D68 RNA shedding in the upper respiratory tract of 12 days after respiratory symptom onset and found that transmission within households, both between children and with parents, occurs. All participants with EV-D68 reported respiratory symptoms, illness duration of EV-D68 was >2 weeks, and most hospitalized children with asthma-like respiratory disease required ICU-level care, highlighting the severity of EV-D68 illness. Notably, this study stresses the importance of recognizing illness early and collecting respiratory specimens promptly in suspected AFM cases. Our findings also underscore the importance of diagnostic advances, such as EV-D68–specific CSF antibody testing, to help detect previous infection when viral RNA is no longer present, confirm cases, and enable targeted treatment when EV-D68–specific therapeutics are approved.
